# Urinary microRNA biomarkers for detecting the presence of esophageal cancer

**DOI:** 10.1038/s41598-021-87925-1

**Published:** 2021-04-20

**Authors:** Yusuke Okuda, Takaya Shimura, Hiroyasu Iwasaki, Shigeki Fukusada, Ruriko Nishigaki, Mika Kitagawa, Takahito Katano, Yasuyuki Okamoto, Tamaki Yamada, Shin-ichi Horike, Hiromi Kataoka

**Affiliations:** 1 Department of Gastroenterology and Metabolism, Nagoya City University Graduate School of Medical Sciences, 1-Kawasumi, Mizuho-cho, Mizuho-ku, Nagoya, 467-8601 Japan; 2 Okazaki Public Health Center, 1-3 Harusaki, Harisaki-cho, Okazaki, 444-0827 Japan; 3 Advanced Science Research Center, Kanazawa University, 13-1 Takaramachi, Kanazawa, 920-8640 Japan

**Keywords:** Biomarkers, Gastroenterology

## Abstract

Esophageal cancer (EC) including esophageal squamous cell carcinoma (ESCC) and adenocarcinoma (EAC) generally exhibits poor prognosis; hence, a noninvasive biomarker enabling early detection is necessary. Age- and sex-matched 150 healthy controls (HCs) and 43 patients with ESCC were randomly divided into two groups: 9 individuals in the discovery cohort for microarray analysis and 184 individuals in the training/test cohort with cross-validation for qRT-PCR analysis. Using 152 urine samples (144 HCs and 8 EACs), we validated the urinary miRNA biomarkers for EAC diagnosis. Among eight miRNAs selected in the discovery cohort, urinary levels of five miRNAs (miR-1273f, miR-619-5p, miR-150-3p, miR-4327, and miR-3135b) were significantly higher in the ESCC group than in the HC group, in the training/test cohort. Consistently, these five urinary miRNAs were significantly different between HC and ESCC in both training and test sets. Especially, urinary miR-1273f and miR-619-5p showed excellent values of area under the curve (AUC) ≥ 0.80 for diagnosing stage I ESCC. Similarly, the EAC group had significantly higher urinary levels of these five miRNAs than the HC group, with AUC values of approximately 0.80. The present study established novel urinary miRNA biomarkers that can early detect ESCC and EAC.

## Introduction

In 2017, esophageal cancer (EC) was the sixth leading cause of cancer-related death worldwide^1^. This malignancy mainly consists of two histological subtypes, namely, esophageal squamous cell carcinoma (ESCC) and esophageal adenocarcinoma (EAC). ESCC accounts for approximately 90% of all ECs worldwide, and the incidence of EAC is rising in several regions, including North America and Europe^2,3^. Despite the advances of medical and surgical treatments of EC, EC remains to exhibit poor prognosis, considering that it is frequently diagnosed at an advanced stage already because patients are generally asymptomatic in the early stage. In Japan, the overall 5-year survival rate of patients with EC is 15–25%^3^, whereas that of patients with EC treated with endoscopic resection and esophagectomy was 84.4% and 55.6%, respectively^4^. Endoscopic examination with biopsy is the golden standard for EC diagnosis; however, endoscopy is not suitable for mass screening because it is highly invasive and expensive. Furthermore, serum tumor markers such as squamous cell carcinoma antigen (SCCA) and carcinoembryonic antigen (CEA), which are clinically used for EC tumor markers, are insufficiently specific, and sensitive for early detecting EC^5,6^. Hence, novel noninvasive biomarkers that can early detect EC are needed so that patients can receive early treatment, with the potential to reduce disease mortality.

MicroRNA (miRNA), which is a class of small noncoding RNAs comprising approximately 18–25 nucleotides, can regulate gene expression by binding to the 3′-UTR of target mRNAs post-transcriptionally, leading to translational inhibition, or promotion of RNA degradation^7^. Dysregulation of miRNAs is reportedly involved in EC tumorigenesis and progression^8–11^. In addition, serum and plasma miRNAs are useful biomarkers of EC^12–14^; however, the expression of urinary miRNAs in patients with EC remains unreported.

Urine is a completely noninvasive sample with huge advantages of easy and high-volume collection and low cost, thereby extremely suitable for the mass screening of healthy individuals. However, no urinary biomarkers have been clinically applied to malignant cases. We have been exploring for urinary biomarkers for a long time and found that urinary protein biomarkers are useful for gastric cancer^15–17^ and colorectal cancer^18^. Moreover, we recently established a urinary miRNA biomarker panel using miR-6807-5p and miR-6856-5p; this panel can early detect gastric cancer^19^. In the present study, we aimed to establish novel urinary miRNA biomarkers for the early detection of EC including ESCC and EAC.

## Methods

### Patients and study design

From September 2012 to August 2018, we collected urine samples at two Japanese institutions (Nagoya City University Hospital and Okazaki Public Health Center). We enrolled males and females aged 20–90 years old. Furthermore, we included patients with EC (EC group) who were histologically confirmed to have carcinoma using endoscopic biopsy or surgical specimen and had no treatment before sample collection. However, those with recurrent EC, a history of neoplasms of any type (within five years), and/or with multiple neoplasms were excluded. Meanwhile, we included healthy control (HC) individuals who were asymptomatic and had no evidence of neoplasms at their annual checkup.

To ensure that this case–control biomarker study is reported accurately and comprehensively, the present study complied with both the REMARK guidelines^20^ and STROBE statement^21^. The ethics committee at each institution (Nagoya City University Hospital Institutional Review Board, and Okazaki Public Health Center Ethical Committee) approved our study protocol (#. 45-12-0013), which conformed to the ethical guidelines of the 1975 Declaration of Helsinki (6th revision, 2008). Each patient provided a written, informed consent before study entry.

### Samples and definition

Urine and serum samples were collected before any treatment for EC, immediately frozen, and stored at − 80 °C until assayed, as previously reported^16–19^. We obtained EC tissues and matched adjacent normal tissues from patients who underwent surgery or endoscopic submucosal dissection (ESD). According to the 7th edition of the Union for International Cancer Control (UICC) TNM classification^22^, the clinical stage was determined by the final pathological diagnosis after resection.

### Cell lines

We utilized a normal esophageal squamous epithelial cell line (Het-1A) (ATCC, Manassas, VA) and three human ESCC cell lines (TE-1, T.T: ATCC) (KYSE-30: ECACC, Salisbury, UK). The cell lines Het-1A, TE-1, T.T, and KYSE-30 were cultured in DMEM containing 10% fatal bovine serum (FBS), RPMI-1640 containing 10% FBS, DMEM/F-12 containing 10% FBS, and RPMI 1640/F-12 containing 2% FBS, respectively, at 37 °C in a humidified atmosphere of 5% CO_2_. To prepare conditioned media (CM) obtained from cell lines, we washed the cells twice with PBS and replaced the culture media with serum-free media. The CM were collected 24 h after media replacement.

### miRNA extraction

We centrifuged the urine samples at 9170 g for 2 min. Then, we extracted the miRNA from 200 μL of urine (600 μL for microarray use) of the supernatant by using miRNeasy Serum/Plasma Kit (QIAGEN, Valencia, CA, USA) according to the manufacturer’s protocol, with the exception that the column was washed thrice using 80% ethanol before total RNAs were eluted in 14 μL of RNase-free water, as previously reported^19^.

Moreover, miRNAs from formalin-fixed paraffin-embedded (FFPE) tissues were extracted using the miRNeasy FFPE Kit (QIAGEN), and total RNAs were eluted in 30 μL of RNase-free water. We also used the miRNeasy Serum/Plasma Kit for extracting miRNAs from CM and then eluted the total RNAs in 14 μL of RNase-free water.

### miRNA microarray assay

We conducted the microarray assay according to a previous report^19^. Briefly, cyanine-3 (Cy3)-labeled cRNA was prepared from urinary RNA from the HC and ESCC groups by using the miRNA Complete Labeling and Hyb Kit (Agilent, Santa Clara, CA, USA) according to the manufacturer’s instructions. The miRNA samples were labeled using T4 RNA ligase and Cy-3-pCp. Cy3-labeled miRNA samples were hybridized in 1 × Hi-PRM hybridization buffer and 1 × GE blocking agent. We hybridized the mixed samples to Agilent Human miRNA Microarrays (G4872A) and scanned the slide on the Agilent DNA Microarray Scanner (G2539A). The scanned images were analyzed with Feature Extraction Software 11.0.1.1 (Agilent).

### Quantitative real-time reverse transcription–polymerase chain reaction (qRT-PCR)

Total RNAs were reverse-transcribed into cDNA by using the TaqMan Advanced miRNA cDNA Synthesis Kit (Applied Biosystems, Foster, CA, USA) according to the manufacture’s protocol. We performed qRT-PCR by using the TaqMan Advanced miRNA Assay (Applied Biosystems) and 7500 Fast Real-time PCR system (Applied Biosystems), with the following thermal cycle sequentially: 20 s at 95 °C and 40 cycles of 3 s at 95 °C and 30 s 60 °C. All reactions were performed twice. Furthermore, the relative gene expression was calculated using the 2^−ΔCt^ method. Internal controls for the qRT-PCR of miRNA in urine and serum were determined by a global mean normalization method with the microarray results^23^. Consequently, miR-4669 and miR-6756-5p were determined as the internal controls for qRT-PCR related to urinary and serum miRNA. For the internal control for the qRT-PCR of miRNA in FFPE tissues and CM, we used RNU6B as the internal control^11^. Supplementary Table S1 lists the miRNA assays used in qRT-PCR.

### Gene functional annotation analysis

We downloaded miRNA-target interactions from the experimentally validated database miRTarBase (version 7.0) (http://mirtarbase.mbc.nctu.edu.tw/). The gene functional annotation analysis was performed using the Database for Annotation, Visualization and Integrated Discovery (DAVID) 6.8 (https://david.ncifcrf.gov/).

### Study flowchart

**Figure 1:**
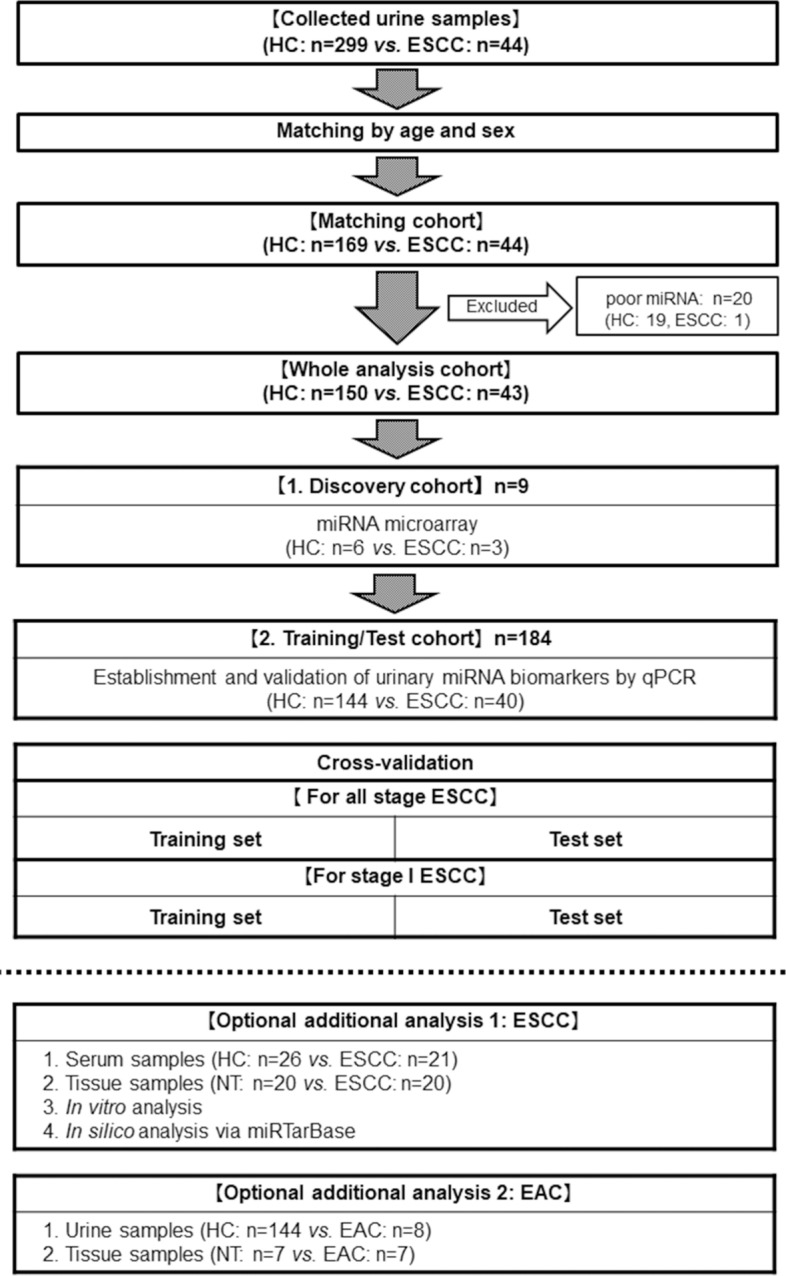
Consort diagram. Optional additional analyses were conducted using all available samples. *HC* healthy control; *ESCC* esophageal squamous cell carcinoma; *qPCR* quantitative polymerase chain reaction, *NT* normal tissue; *EAC* esophageal adenocarcinoma.

In total, 343 participants comprising 299 HCs and 44 ESCCs were enrolled for this urinary biomarker study of ESCC. Propensity score (PS), which included age and sex, was estimated using a logistic regression model. We randomly matched with case-to-control ratio = 1:4, using the nearest-neighbor method within a caliper of width of 0.2 of the SD of the logit of the PS. C-statistic of the PS matching model was 0.742. After case matching and exclusion of 20 urine samples with poor miRNA quality, 193 patients (150 HCs and 43 ESCCs) were ultimately selected as the whole analysis cohort. This entire cohort was randomly divided into two by the computer: 9 individuals (6 HCs and 3 ESCCs) in the discovery cohort and 184 individuals (144 HCs and 40 ESCCs) in the training/test cohort. In the discovery cohort, potential urinary miRNA biomarkers for ESCC diagnosis were identified by miRNA microarray analysis; then, in the training/test cohort, qRT-PCR analysis confirmed, and established the urinary miRNA biomarkers. The established biomarkers were then validated by internal cross-validation analysis for all stage ESCC and stage I ESCC in training and test sets, respectively. Additionally, we analyzed all available serum (26 HCs and 21 ESCCs) and tissue (20 ESCC tissues) miRNAs and also performed in vitro and in silico analyses. Finally, we validated the urinary miRNA biomarkers for EAC diagnosis, using all available 152 urine (144 HCs and 8 EACs) and 7 EAC tissue samples (Fig. 1).

### Statistical analysis

Quantitative variables were described with median and interquartile range [IQR] and analyzed using the Mann–Whitney U test. Categorical variables were analyzed using chi-square test or the Fisher’s exact probability test, as appropriate. Correlations were evaluated using Spearman’s rank method with a coefficient (*r*). Furthermore, the area under the curve (AUC) for each biomarker was calculated by receiver operating characteristic (ROC) curve analysis, and the representative value was shown as the AUC value with 95% confidence interval (CI). Sensitivity and specificity of each biomarker were determined by the optimal cutoff value. To evaluate the generalization of the established biomarkers, we performed internal validation using the *k*-fold cross-validation method (*k* = 5)*.* The data set of the training/validation cohort was divided into five subsets in which four were used for the training set and one was used for the test set in each analysis. The ROC analyses for both sets were repeated five times. Finally, the AUC represented an average value across all analyses, and 95% CI was estimated by bootstrap method that resampled the model 1000 times. All statistics were calculated using the SPSS Statistics version 25 (IBM, Tokyo, Japan) and R software (https://www.r-project.org/). Two-sided *P* values < 0.05 were considered statistically significant.

## Results

### Patients

Table 1 summarizes the overall patient characteristics. Age, sex, and serum creatinine were not significantly different between the HC and ESCC groups. Stage I and T1 stage were found in 37.2% and 48.8% of ESCCs, respectively.

**Table 1:** Characteristics of the whole analysis cohort.

	HC (n = 150)	ESCC (n = 43)	*P* value	d
Median age [IQR] (years)	69 [63–74]	70 [65–75]	0.072^†^	0.34
**Sex**			1.000^‡^	0.03
Female	15 (10.0%)	4 (9.3%)		
Male	135 (90.0%)	39 (90.7%)		
Median serum creatinine [IQR] (mg/dl)	0.84 [0.76–0.94]	0.81 [0.70–0.99]	0.592^†^	0.06
**Tumor location**				
Cervical		2 (4.7%)		
Thoracic		39 (90.7%)		
Abdominal		2 (4.7%)		
**TNM stage**				
I		16 (37.2%)		
II		4 (9.3%)		
III		12 (27.9%)		
IV		11 (25.6%)		
**Differentiation (squamous cell carcinoma)**				
Well to moderately		39 (90.7%)		
Poorly		3 (7.0%)		
Basaloid		1 (2.3%)		
**T stage**				
T1		21 (48.8%)		
T2		1 (2.3%)		
T3		10 (23.3%)		
T4		11 (25.6%)		

*HC* healthy control; *ESCC* esophageal squamous cell carcinoma; *IQR* interquartile range; *d* standardized difference.

^†^Mann–Whitney U test.

^‡^Fisher’s exact probability test.

### Comprehensive analysis with miRNA microarray

To identify urinary miRNA biomarker candidates, we first interrogated a microarray-based miRNA expression profiling comparing the urinary miRNA expression levels of the HC group with those of the ESCC group, in the discovery cohort. Seventy miRNAs (22 up-regulated and 48 down-regulated miRNAs) were differentially expressed between the two groups (absolute fold change > 2.0, *P* < 0.05) (Supplementary Fig. S1). Among these miRNAs, we selected eight miRNAs which were stably expressed in both microarray analysis and preliminary qRT-PCR analysis for the succeeding step.

### Establishment and validation of urinary miRNA biomarkers using qRT-PCR

**Figure 2:**
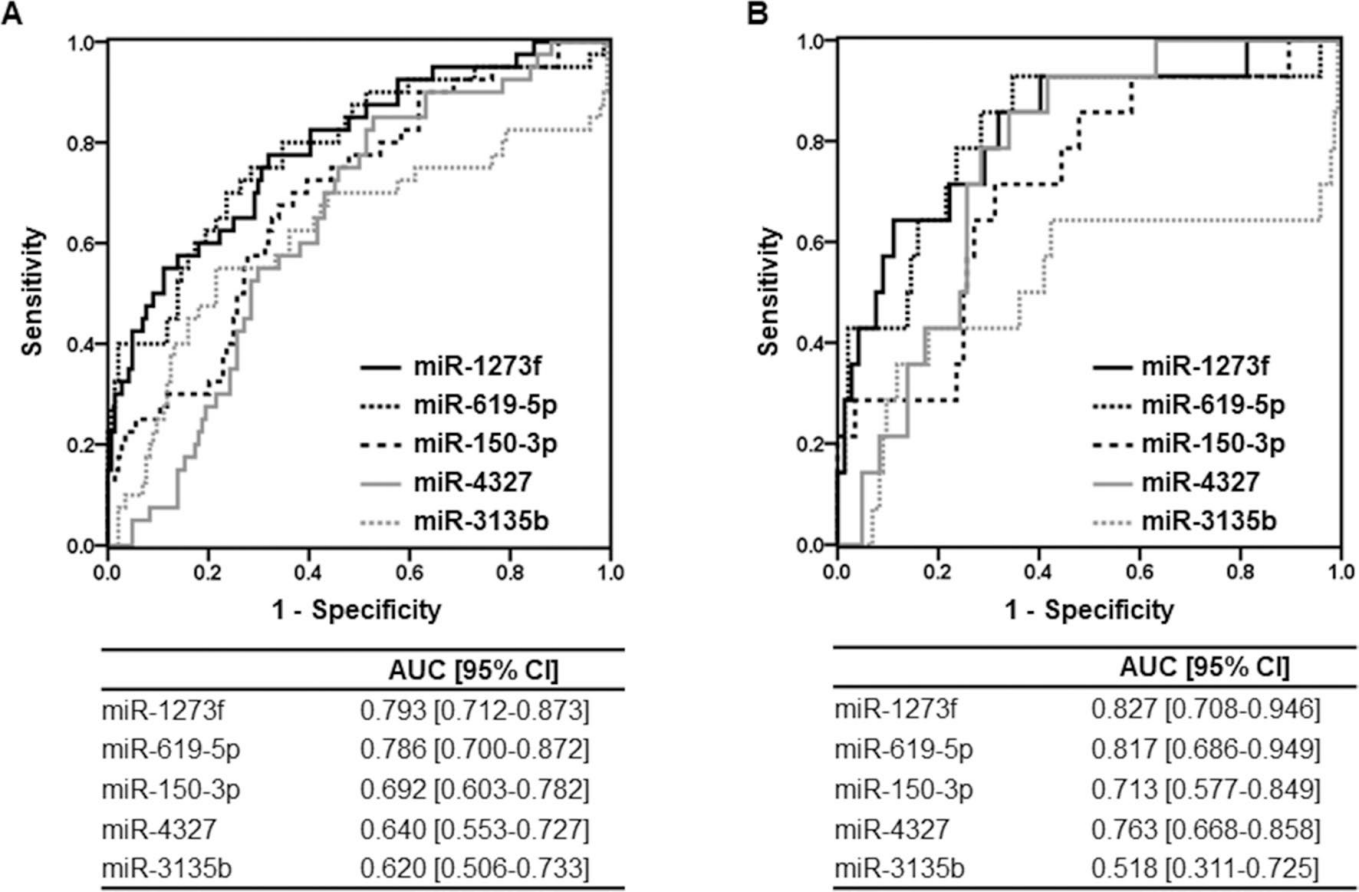
Receiver operating characteristic curves in the training/test cohort. (**A**) HC vs. all stage ESCC, (**B**) HC vs. stage I ESCC. Receiver operating characteristic curves were obtained from five urinary miRNAs. *AUC* area under the curve; *95% CI* 95% confidence interval.

Next, using the qRT-PCR assay, we evaluated the urinary expression levels of eight miRNAs (miR-1273f, miR-619-5p, miR-150-3p, miR-4327, miR-3135b, miR-5585-3p, miR-6875-5p, and miR-345-3p) in the training/test cohort. The urinary levels of miR-1273f, miR-619-5p, miR-150-3p, miR-4327, and miR-3135b were significantly higher in the ESCC group than in the HC group (miR-1273f, *P* < 0.001; miR-619-5p, *P* < 0.001; miR-150-3p, *P* < 0.001; miR-4327, *P* = 0.007; miR-3135b, *P* = 0.021; Table 2). Subsequently, we conducted internal cross-validation analysis in the training and test sets according to the *k*-fold cross-validation method (*k* = 5). These five urinary miRNAs were consistently significantly different between HC and all stage ESCC (AUC: miR-1273f: 0.792, 0.788; miR-619-5p: 0.786, 0.786; miR-150-3p: 0.692, 0.696; miR-4327: 0.639, 0.625; miR-3135b: 0.620, 0.626 in the training and test sets, respectively) (Table 3). In total, these urinary miRNAs were also significantly different between the HC and ESCC groups in ROC analysis (Fig. 2A). Urinary miR-1273f and miR-619-5p were the top two qualified biomarkers for ESCC diagnosis, with 82.5% and 80.0% sensitivity and 59.0% and 65.3% specificity, respectively.

**Table 2:** Urinary levels of miRNAs.

	Median 2^−ΔCT^ (× 10^–2^) [IQR]		*P* value	Median 2^−ΔCT^ (× 10^–2^) [IQR]	*P* value
	HC	All stage ESCC	HC *vs.* All Stages	Stage I ESCC	HC *vs.* Stage I
miR-1273f	3.313 [1.694–7.899]	14.014 [5.482–45.629]	< 0.001	15.257 [6.725–45.494]	< 0.001
miR-619-5p	23.183 [12.112–42.318]	73.644 [34.289–188.674]	< 0.001	73.183 [44.617–165.866]	< 0.001
miR-150-3p	0.594 [0.307–1.731]	1.517 [0.654–3.116]	< 0.001	1.633 [0.692–4.037]	0.009
miR-4327	29.680 [14.314–84.168]	64.618 [30.404–121.001]	0.007	89.331 [62.138–172.789]	0.001
miR-3135b	99.363 [35.556–235.372]	263.782 [42.933–589.915]	0.021	146.608 [9.591–613.165]	0.821
miR-5585-3p	1.416 [0.563–3.674]	2.319 [0.785–5.535]	0.183	2.426 [0.377–10.734]	0.337
miR-6875-5p	6.538 [3.759–17.432]	7.785 [3.969–11.871]	0.629	9.517 [3.962–17.756]	0.807
miR-345-3p	0.081 [0.017–0.263]	0.105 [0.026–0.216]	0.562	0.096 [0.019–0.220]	0.883

*HC* healthy control; *ESCC* esophageal squamous cell carcinoma; *IQR* interquartile range.

**Table 3:** Internal cross-validation of urinary miRNA biomarker.

	Training set Mean AUC [95% CI]	Test set Mean AUC [95% CI]
**All stage ESCC**		
miR-1273f	0.792 [0.777–0.811]	0.788 [0.713–0.850]
miR-619-5p	0.786 [0.771–0.801]	0.786 [0.727–0.846]
miR-150-3p	0.692 [0.678–0.709]	0.696 [0.628–0.760]
miR-4327	0.639 [0.627–0.653]	0.625 [0.589–0.664]
miR-3135b	0.620 [0.602–0.635]	0.626 [0.556–0.703]
**Stage I ESCC**		
miR-1273f	0.826 [0.803–0.848]	0.819 [0.727–0.910]
miR-619-5p	0.817 [0.794–0.852]	0.823 [0.696–0.921]
miR-150-3p	0.713 [0.680–0.753]	0.705 [0.563–0.822]
miR-4327	0.749 [0.729–0.766]	0.733 [0.661–0.796]
miR-3135b	0.520 [0.490–0.553]	0.558 [0.399–0.714]

Mean AUC with 95% CI was estimated by bootstrap method that resampled the model 1000 times.

*ESCC* esophageal squamous cell carcinoma; *AUC* area under the curve; *95% CI* 95% confidence interval.

Importantly, the urinary levels of miR-1273f, miR-619-5p, miR-150-3p, and miR-4327 were significantly higher in the stage I ESCC group than in the HC group (miR-1273f, *P* < 0.001; miR-619-5p, *P* < 0.001; miR-150-3p, *P* = 0.009; miR-4327, *P* = 0.001; Table 2). These urinary miRNAs consistently exhibited excellent diagnostic performance for stage I ESCC in both training and test sets (AUC in the training and test sets: miR-1273f: 0.826, 0.819; miR-619-5p: 0.817, 0.823; miR-150-3p: 0.713, 0.705; miR-4327: 0.749, 0.733) (Table 3). In total, these four urinary miRNAs were significantly different between HC and stage I ESCC (Fig. 2B). Specifically, urinary miR-1273f and miR-619-5p were 92.9% and 92.9% sensitive and 59.0% and 65.3% specific, respectively, for detecting stage I ESCC.

The urinary levels of miR-1273f, miR-619-5p, miR-150-3p, and miR-3135b had no significant correlation with disease stage, although miR-4327 negatively correlated with disease stage (*r* =  − 0.435) (Supplementary Fig. S2).

### Additional validation analysis using serum, tissue samples, and cell lines

To further validate the established urinary miRNA biomarkers, we conducted additional analyses using serum and tissue samples and in vitro analysis. The serum levels of the five miRNAs (miR-1273f, miR-619-5p, miR-150-3p, miR-4327, and miR-3135b) were compared between 26 HCs and 21 ESCCs. Patient characteristics were not significantly different between the two groups. Despite significant differences in urine samples, the serum levels of all five miRNAs had no significant differences between the ESCC and HC groups (Supplementary Table S2). Regarding tissue miRNA analysis, 20 ESCC samples were available for miRNA extraction, of which 11 patients underwent surgery and nine patients underwent ESD, and 13 patients (65.0%) were still in stage I. The expression levels of miR-1273f, miR-619-5p, miR-150-3p, and miR-4327 tended to be higher in the ESCC tissues than those in the adjacent normal tissues, but not significant (Supplementary Fig. S3). Despite no significance, these tendencies were also observed in the analysis using 13 stage I ESCC tissues and adjacent normal tissues (Supplementary Fig. S4).

**Figure 3:**
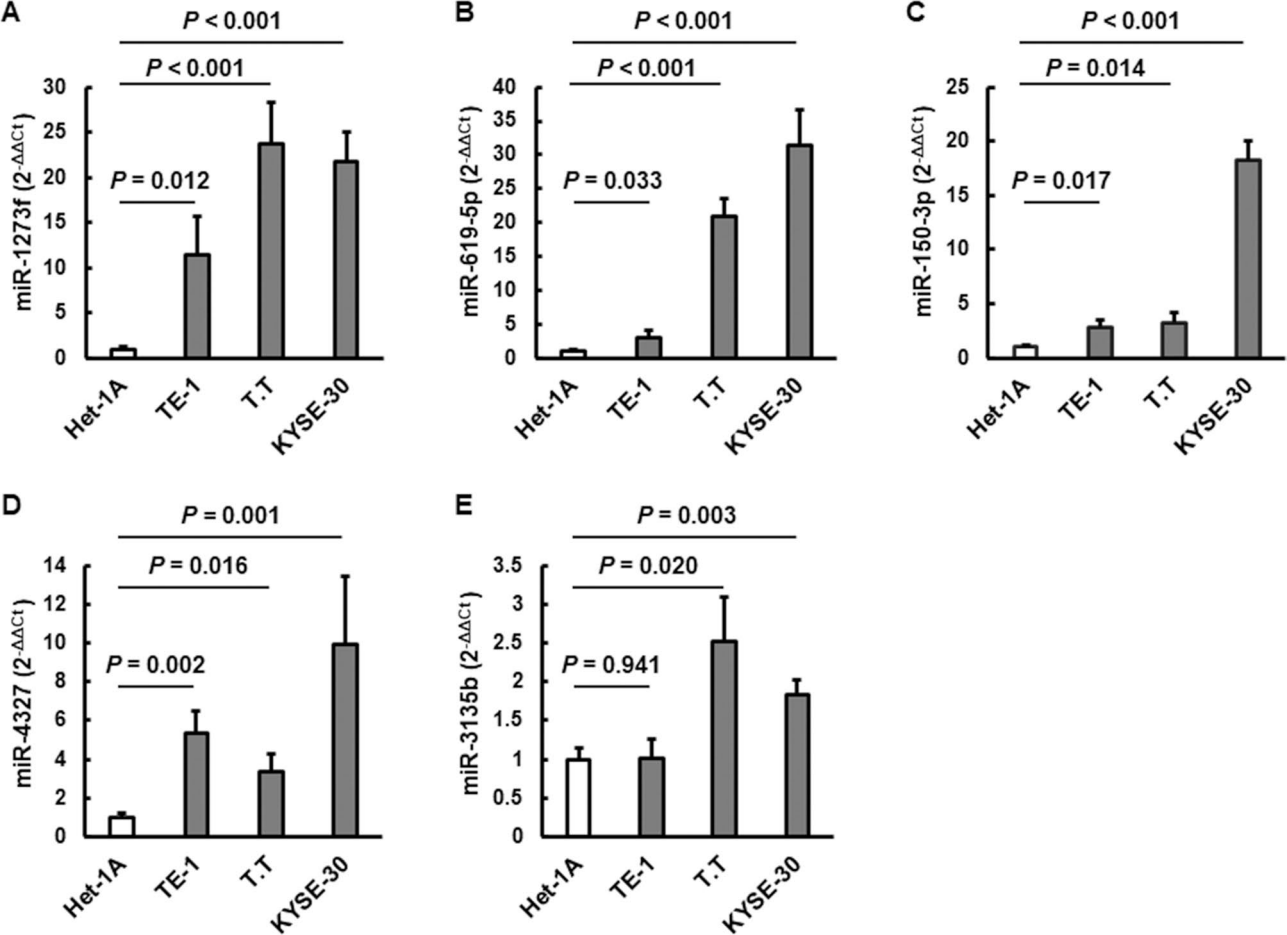
Expression levels of miRNAs in the conditioned media from esophageal cell lines. (**A**) miR-1273f, (**B**) miR-619-5p, (**C**) miR-150-3p, (**D**) miR-4327, (**E**) miR-3135b. The expression levels of miRNAs in the three human esophageal squamous cell carcinoma cell lines (TE-1, T.T, and KYSE-30) are shown by its relative ratio to those in a normal esophageal squamous epithelial cell line (Het-1A). Data are expressed as the mean ± SD.

In addition, using the CM from normal esophageal and ESCC cell lines, we investigated whether these miRNAs were released from ESCC. As shown in Fig. 3, these miRNAs were significantly more abundant in the CM from the ESCC cell lines than those in the CM from the normal esophageal cell line.

### Esophageal adenocarcinoma analysis

**Figure 4:**
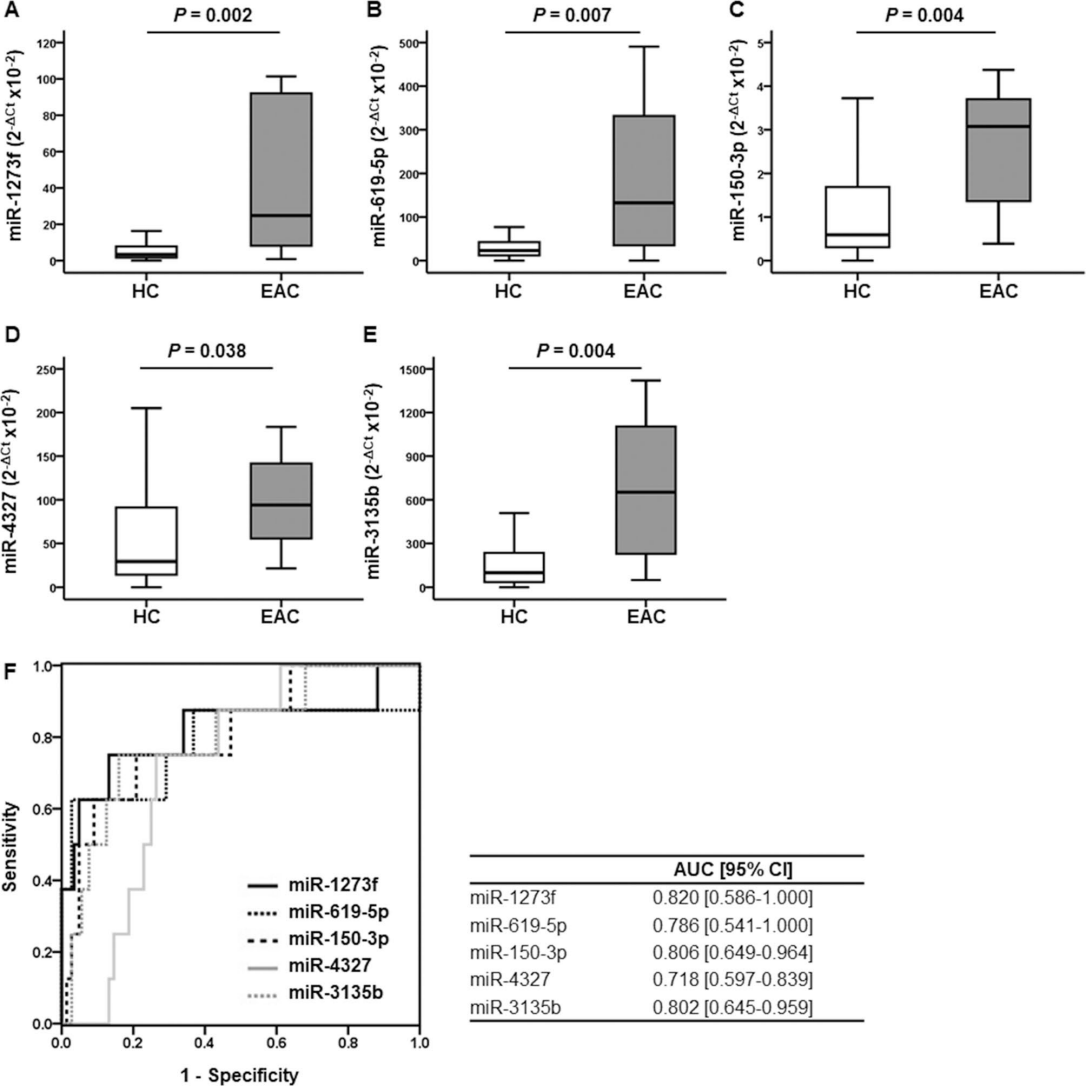
Urinary expression levels of miRNAs in the esophageal adenocarcinoma cohort. (**A**) miR-1273f., (**B**) miR-619-5p, (**C**) miR-150-3p, (**D**) miR-4327, (**E**) miR-3135b. Data were analyzed by Mann–Whitney U test. (**F**) Receiver operating characteristic curves. Receiver operating characteristic curves were obtained from urinary miRNAs (HC vs. EAC). *EAC* esophageal adenocarcinoma; *AUC* area under the curve; *95% CI* 95% confidence interval.

Moreover, we investigated whether the established urinary miRNA biomarkers for ESCC can be applied to EAC. In the EAC biomarker study, 152 participants consisting of 144 HCs and 8 EACs were analyzed. Patient characteristics were similar between two groups, and 37.5% of patients with EAC were in stage I (Supplementary Table S3). Consistent with ESCC, the EAC group had significantly higher urinary levels of all the five miRNAs than the HC group (miR-1273f: median 2^−ΔCT^ [× 10^−2^], 24.834 vs. 3.313, *P* = 0.002; miR-619-5p: median, 132.526 vs. 23.183, *P* = 0.007; miR-150-3p: median, 3.073 vs. 0.594, *P* = 0.004; miR-4327: median, 94.140 vs. 29.680, *P* = 0.038; miR-3135b: median, 651.224 vs. 99.363, *P* = 0.004; Fig. 4A–E). Moreover, these five urinary miRNAs were significantly different between HC and EAC, with excellent AUCs (Fig. 4F).

We also tested the tissue expression levels of miRNA for 7 EAC tissues (5: surgery, 2: ESD) that were available for miRNA extraction. Despite having no significance, the expression levels of all the five miRNAs tended to be higher in the EAC tissues than in the adjacent normal tissues (Supplementary Fig. S5).

### In silico analysis

Finally, we conducted gene functional annotation analysis to explore the functions of the established urinary miRNAs. In miRTarBase, 295, 425, 40, 96, and 394 genes were listed as the targets of miR-1273f, miR-619-5p, miR-150-3p, miR-4327, and miR-3135b, respectively. The gene functional annotation analysis demonstrated that approximately 45–65% of the target genes for each miRNA possess functions associated with phosphoprotein (Supplementary Fig. S6).

## Discussion

This study is the first to demonstrate urinary miRNA biomarkers for diagnosing EC. Given that circulating miRNAs in body fluids are protected from degradation by binding to RNA-binding proteins such as lipoproteins or being contained in extracellular vesicles such as microvesicles and exosomes^24–26^, these miRNAs are potential candidates for minimally invasive biomarkers for cancer diagnosis^27,28^. Urine sample is one of the most attractive biomarker samples because it is noninvasive and abundant. The only possible disadvantage of urine is that the urine concentration fluctuates depending on the time of collection, however, it can be overcome by normalization with internal controls. The current study identified five urinary miRNAs (miR-1273f, miR-619-5p, miR-150-3p, miR-4327, and miR-3135b) as potential diagnostic biomarkers for ESCC. Among them, four (miR-1273f, miR-619-5p, miR-150-3p, and miR-4327) could efficiently detect even in stage I ESCC. Interestingly, the urinary levels of these miRNAs did not correlate with disease stage, suggesting that these urinary levels increased from stage I and then plateaued after subsequent disease progression. Especially, miR-1273f and miR-619-5p exhibited excellent diagnostic performance for early ESCC detection, with AUC values beyond 0.80 individually. Sensitivities of miR-1273f and miR-619-5p were 82.5% and 80.0% in all stage and 92.9% and 92.8% in stage I ESCC, which showed much better detection rates compared to those of anti-p53 antibodies (around 25% to 30% in all stage ESCC and around 20% in stage I ESCC)^29,30^. Given that the diagnostic performance of these urinary miRNA biomarkers was clearly better than currently used tumor markers, these urinary miRNAs may have contributed to disease curability. Notably, these five urinary miRNAs (miR-1273f, miR-619-5p, miR-150-3p, miR-4327, and miR-3135b) were also diagnostic biomarkers for EAC, with excellent AUC values in the present study.

Although the functional significances of five miRNAs remain unknown, the correlation between these miRNAs and various cancers has been reported in some studies. The expression of miR-1273f was significantly elevated within exosomes released by hypoxic hepatocellular carcinoma cells than that by normoxic cells, and it could activate the Wnt/β-catenin signaling pathway^31^. Meanwhile, the exosome and plasma levels of miR-619-5p were significantly higher in non-small cell lung cancer^32^ and prostate cancer cases^33^ than in the HCs, whereas the expression level of miR-619-5p was significantly lower in colorectal cancer tissues than in normal tissues^34^. Furthermore, miR-150-3p reportedly has diverse expression patterns, in which miR-150 was up-regulated in gastric cancer tissues^35^ but down-regulated in lung adenocarcinoma tissues compared with that in normal tissues^36^. In contrast, a previous small sample study with 20 ESCC cases reported that the ESCC tissues had a significantly down-regulated expression level of miR-150-3p than the normal tissues^37^. This inconsistent result with our study is probably caused by the study sample size. Moreover, reports related to miR-4327 are still currently unavailable, but it may be related to early tumorigenesis because of negative correlation with disease stage in this study. As for miR-3135b, it was down-regulated in colorectal cancer cell lines and clinical tumors; when it is overexpressed, cell proliferation is significantly inhibited^38^. Considering the abovementioned existing contradictive results, further studies are thereby needed to elucidate the oncological function of these five miRNAs. In addition, it is interesting why these urinary miRNAs were elevated in both ESCC and EAC. Although the exact mechanism is still unknown, there are several possible reasons. First, the anatomical reason of the esophagus is presumed. Regardless of histology, tumorigenesis and differentiation of the esophageal epithelium may be associated with these miRNAs. Second, these miRNAs may be involved in non-specific functions of tumorigenesis regardless of histology, such as cell proliferation, angiogenesis and squamous epithelial differentiation. As analyzed by in silico analysis, the target genes of the established urinary miRNAs were strongly associated with phosphoprotein. The importance of phosphorylation at the molecular level is particularly related to signaling pathways involved in the etiology of various cancers.

Presumably, our established urinary miRNAs originated from cancer cells and were excreted via urine according to the present results of in vitro and tissue expression analyses. However, these miRNAs were not significant biomarkers in the serum samples. The previous study analyzed the expression of miRNAs in 12 body fluids, including plasma and urine, and reported that the miRNA profiles vary among the fluids. In particular, the plasma miRNA differed from that of most of other body fluids, indicating an extensive “filtering” process separating the plasma from other body fluid types^27^. Compared with urine samples, blood samples contain numerous proteins, nucleic acids, exosomes, and small molecules. These complex components in blood samples might result in nonspecific signals, leading to severe interference to the real target. Notably, the accuracy of our established urinary miRNA biomarkers was similar to that of the previously reported serum/plasma miRNA biomarkers studies for ESCC^12,13^ and EAC^39^, with the AUC range of 0.754–0.846. However, the current urinary miRNA biomarkers were noninvasive compared with blood-based biomarkers.

There are a few limitations in the present study. First, sample size is relatively small. However, the present study cohort contained 37% of stage I ESCC and EAC patients. Moreover, the diagnostic performance of urinary miRNA biomarkers was validated by cross-validation analysis. From these results, the current urinary miRNA biomarkers might represent solid evidence with novel insight. Second, although no significant differences were noted, balances between 2 groups for age and serum creatinine is not still so good after PS matching with standardized difference > 0.1. However, since urinary levels of all 5 miRNAs were not correlated with age and serum creatinine level (|*r*|< 0.2), these tiny unbalances would be negligible. Third, there are no data related to drinking and smoking habits in this study. In order to resolve all these limitations, we are going to start a prospective cohort study soon for the future clinical application.

In conclusion, the present study established novel and reliable urinary miRNA biomarkers that enable early detection of ESCC and EAC.

## Supplementary Information

Supplementary Information.

## Data Availability

The datasets used and/or analysed during the current study are available from the corresponding author on reasonable request.
