# Supplementary material for: Urinary microRNA biomarkers for detecting the presence of esophageal cancer
Source: Sci Rep. 2021 Apr 20;11:8508. doi: 10.1038/s41598-021-87925-1 (PMC8058072; doi:10.1038/s41598-021-87925-1)
Supplement: Supplementary file 1 — Supplementary Information. [file 41598_2021_87925_MOESM1_ESM.pdf]

Supplementary Information

Urinary microRNA biomarkers for detecting the presence of esophageal cancer

Yusuke Okuda[1], Takaya Shimura[1*], Hiroyasu Iwasaki[1], Shigeki Fukusada[1], Ruriko Nishigaki[1], Mika Kitagawa[1], Takahito Katano[1], Yasuyuki Okamoto[1], Tamaki Yamada[2], Shin-ichi Horike[3], Hiromi Kataoka[1]

[1] Department of Gastroenterology and Metabolism, Nagoya City University Graduate School of Medical Sciences, 1-Kawasumi, Mizuho-cho, Mizuho-ku, Nagoya 467-8601, Japan

[2] Okazaki Public Health Center, 1-3 Harusaki, Harisaki-cho, Okazaki 444-0827, Japan

[3] Advanced Science Research Center, Kanazawa University, 13-1 Takaramachi, Kanazawa 920-8640, Japan

Supplementary Table S1-3

Supplementary Fig. S1-6

Supplementary Table S1. TaqMan Advanced miRNA Assays for qRT-PCR

Assay name	Assay ID	Mature miRNA sequence
hsa-miR-4669	478925_mir	UGUGUCCGGGAAGUGGAGGAGG
hsa-miR-6756-5p	480284_mir	AGGGUGGGGCUGGAGGUGGGGCU
hsa-miR-1273f	478679_mir	GGAGAUGGAGGUUGCAGUG
hsa-miR-619-5p	479103_mir	GCUGGGAUUACAGGCAUGAGCC
hsa-miR-150-3p	478721_mir	CUGGUACAGGCCUGGGGGACAG
hsa-miR-4327	478894_mir	GGCUUGCAUGGGGGACUGG
hsa-miR-3135b	478011_mir	GGCUGGAGCGAGUGCAGUGGUG
hsa-miR-5585-3p	480128_mir	CUGAAUAGCUGGGACUACAGGU
hsa-miR-6875-5p	480501_mir	UGAGGGACCCAGGACAGGAGA
hsa-miR-345-3p	478833_mir	GCCCUGAACGAGGGGUCUGGAG

Supplementary Table S2. Serum levels of miRNAs

	Univariate analysis		
	Median $2^{-\Delta CT}$ [IQR]		***P* value**
	**HC**	**ESCC**	
**miR-1273f**	3.731 [2.538-5.110]	3.832 [2.441-4.630]	0.748†
**miR-619-5p**	25.536 [16.026-33.506]	27.602 [19.068-38.461]	0.748†
**miR-150-3p**	0.251 [0.202-0.350]	0.324 [0.203-0.469]	0.507†
**miR-4327**	4.687 [3.380-6.267]	4.211 [3.216-5.503]	0.460†
**miR-3135b**	20.995 [13.644-34.763]	22.516 [9.052-26.266]	0.578†

†, Mann-Whitney U test

HC, healthy control; ESCC, esophageal squamous cell carcinoma; IQR, interquartile range

Supplementary Table S3. Characteristics of esophageal adenocarcinoma

	**HC (n=144)**	**EAC (n=8)**	***P* value**
Median age [IQR] (years)	69 [63-74]	66 [62-74]	0.722†
Sex			1.000‡
Female	13 (9.0%)	0 (0%)	
Male	131 (91.0%)	8 (100%)	
Median serum creatinine [IQR] (mg/dl)	0.84 [0.76-0.94]	0.93 [0.70-1.01]	0.460†
Tumor location			
Cervical		0 (0%)	
Thoracic		3 (37.5%)	
Abdominal		5 (62.5%)	
TNM Stage			
I		3 (37.5%)	
II		2 (25.0%)	
III		2 (25.0%)	
IV		1 (12.5%)	
Differentiation (adenocarcinoma)			
Well to moderately		6 (75.0%)	
Poorly		2 (25.0%)	
T stage			
T1		2 (25.0%)	
T2		2 (25.0%)	
T3		3 (37.5%)	
T4		1 (12.5%)	

†, Mann-Whitney U test

‡, Fisher's exact probability test

HC, healthy control; EAC, esophageal adenocarcinoma; IQR, interquartile range

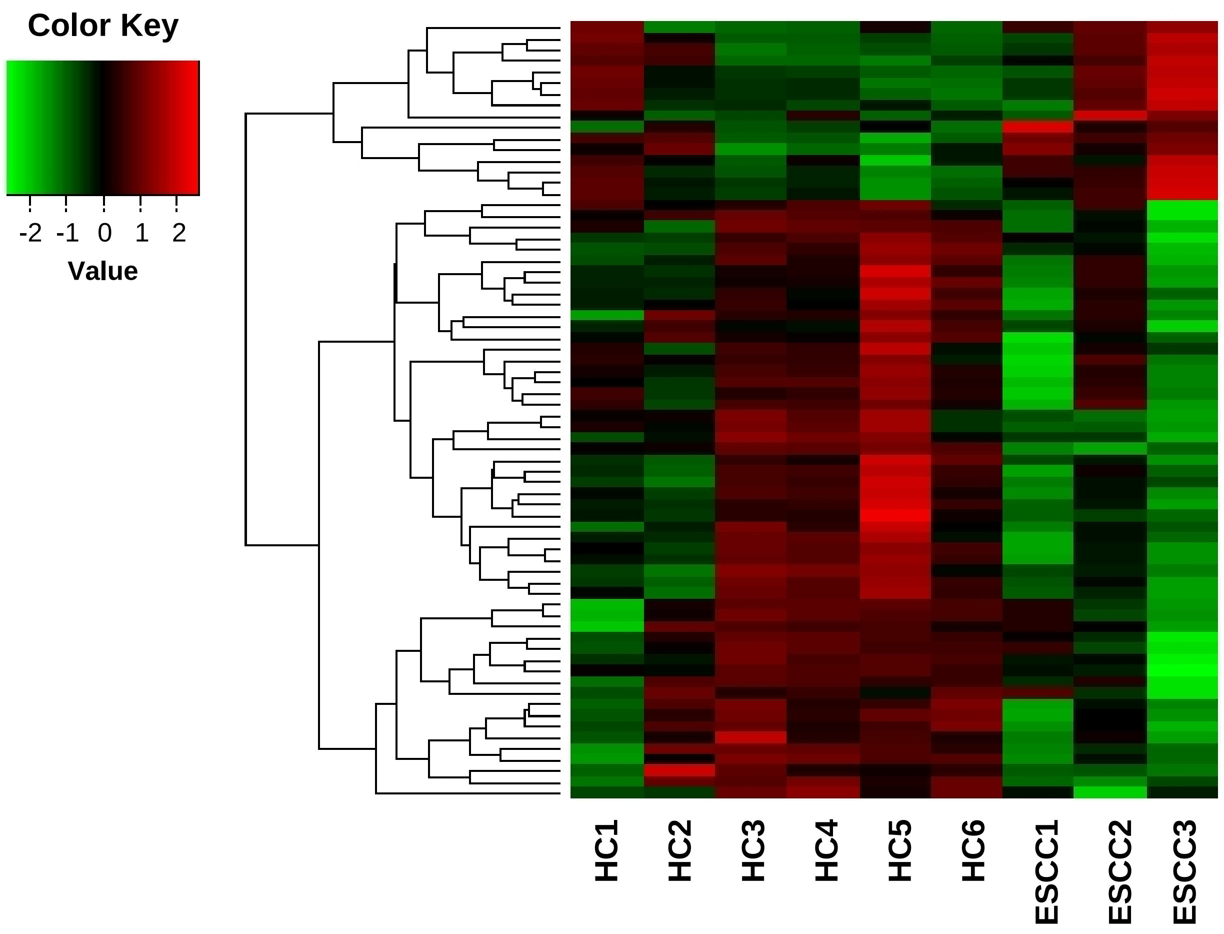

Supplementary Fig. S1. miRNA array in the discovery cohort.
The heat map of the differentially expressed urinary miRNAs between HC and ESCC group. HC, healthy control; ESCC, esophageal squamous cell carcinoma. The map was generated using the gplots package (version 3.0.1.1) in R.

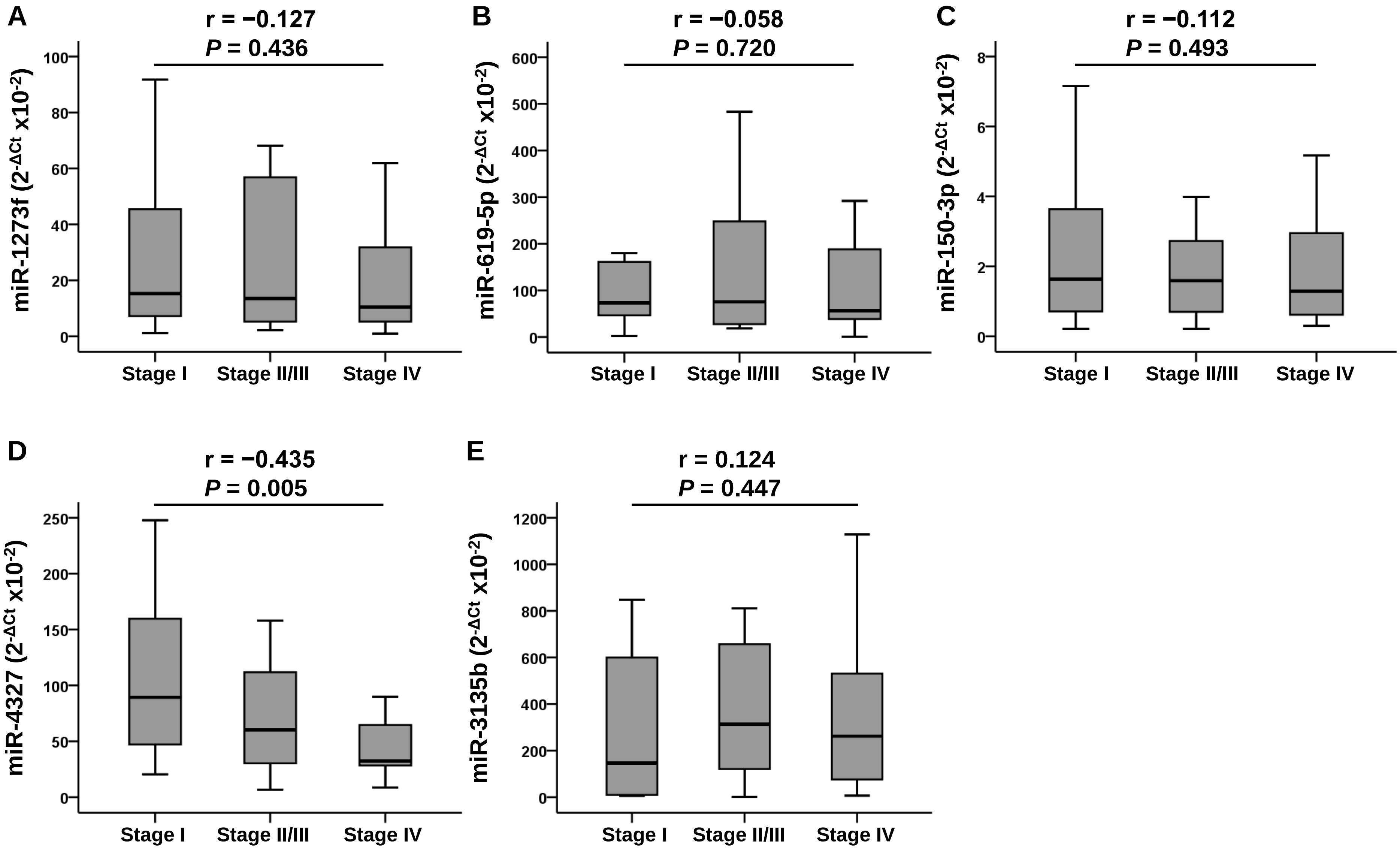

Supplementary Fig. S2. Correlation between urinary levels of miRNAs and disease stage of esophageal squamous cell carcinoma. A. miR-1273f. B. miR-619-5p. C. miR-150-3p. D. miR-4327. E. miR-3135b.
Data were analyzed using the Spearman rank correlation.

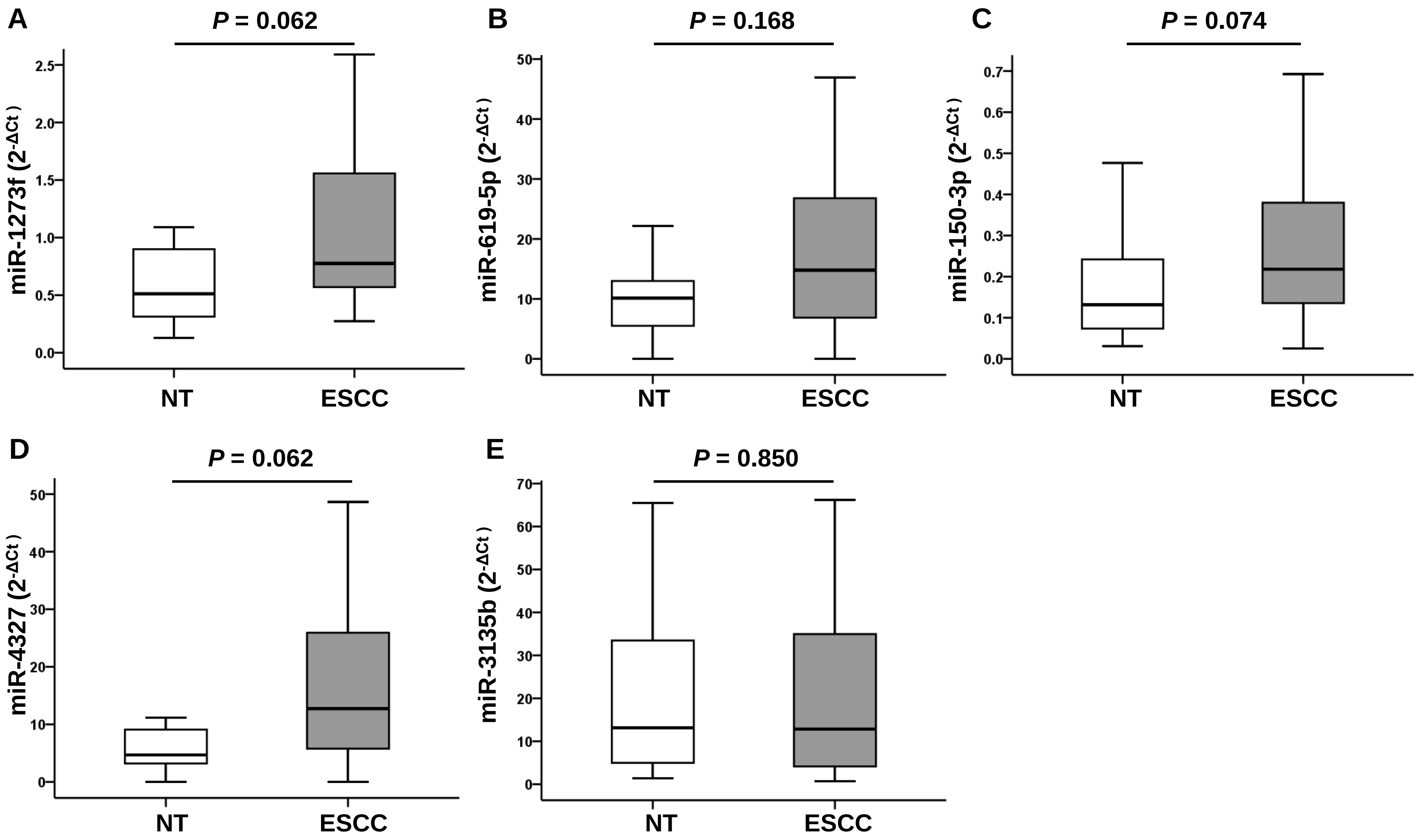

Supplementary Fig. S3. miRNA expression in the esophageal squamous cell carcinoma and adjacent normal tissues. A. miR-1273f. B. miR-619-5p. C. miR-150-3p. D. miR-4327. E. miR-3135b.

Data were analyzed using Mann-Whitney U test. (NT, N =20; ESCC, N = 20)

NT, normal tissue; ESCC, esophageal squamous cell carcinoma

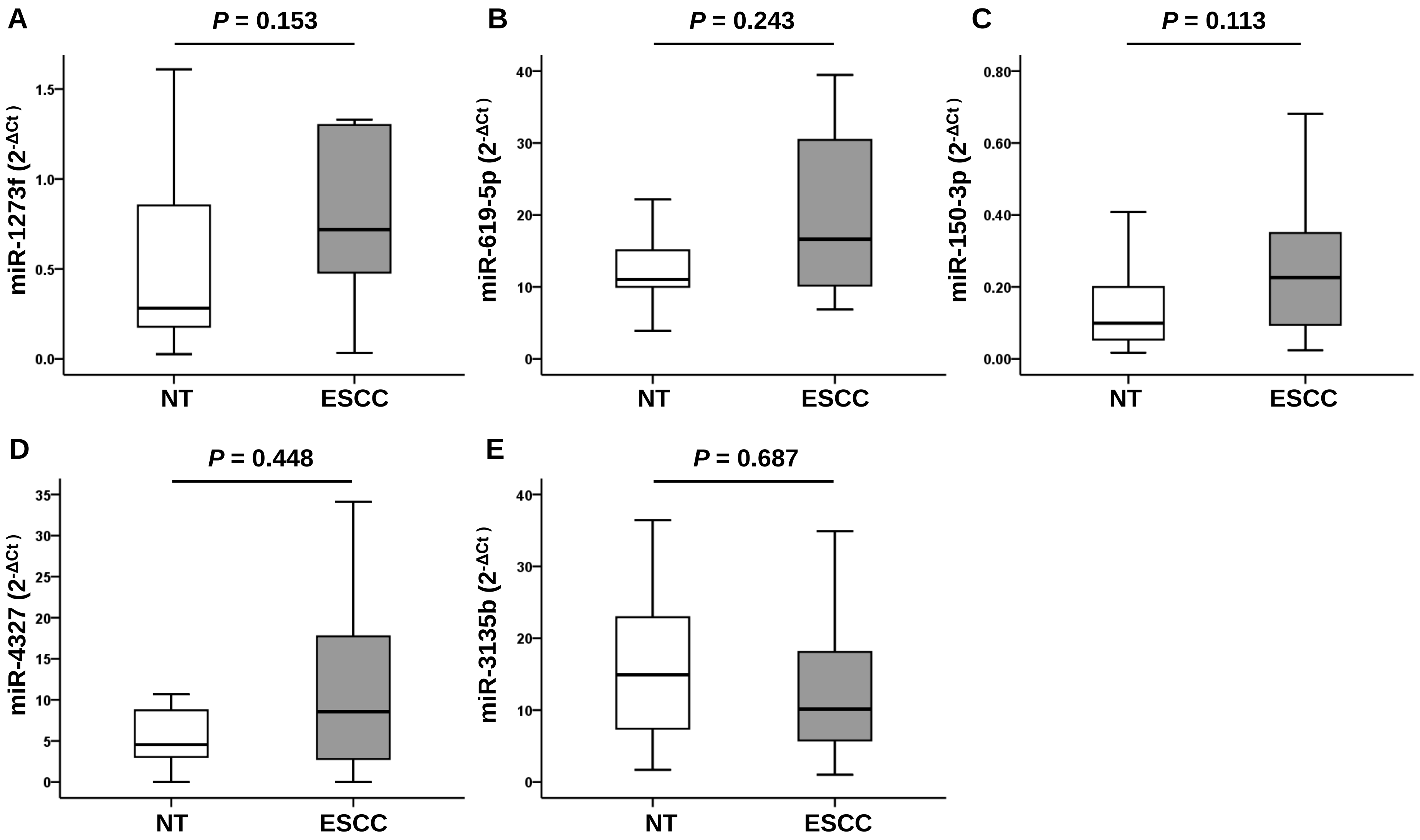

Supplementary Fig. S4. miRNA expression in the stage I esophageal squamous cell carcinoma and adjacent normal tissues.
A. miR-1273f. B. miR-619-5p. C. miR-150-3p. D. miR-4327. E. miR-3135b.
Data were analyzed using Mann-Whitney U test. (NT, N =13; ESCC, N = 13)
NT, normal tissue; ESCC, esophageal squamous cell carcinoma

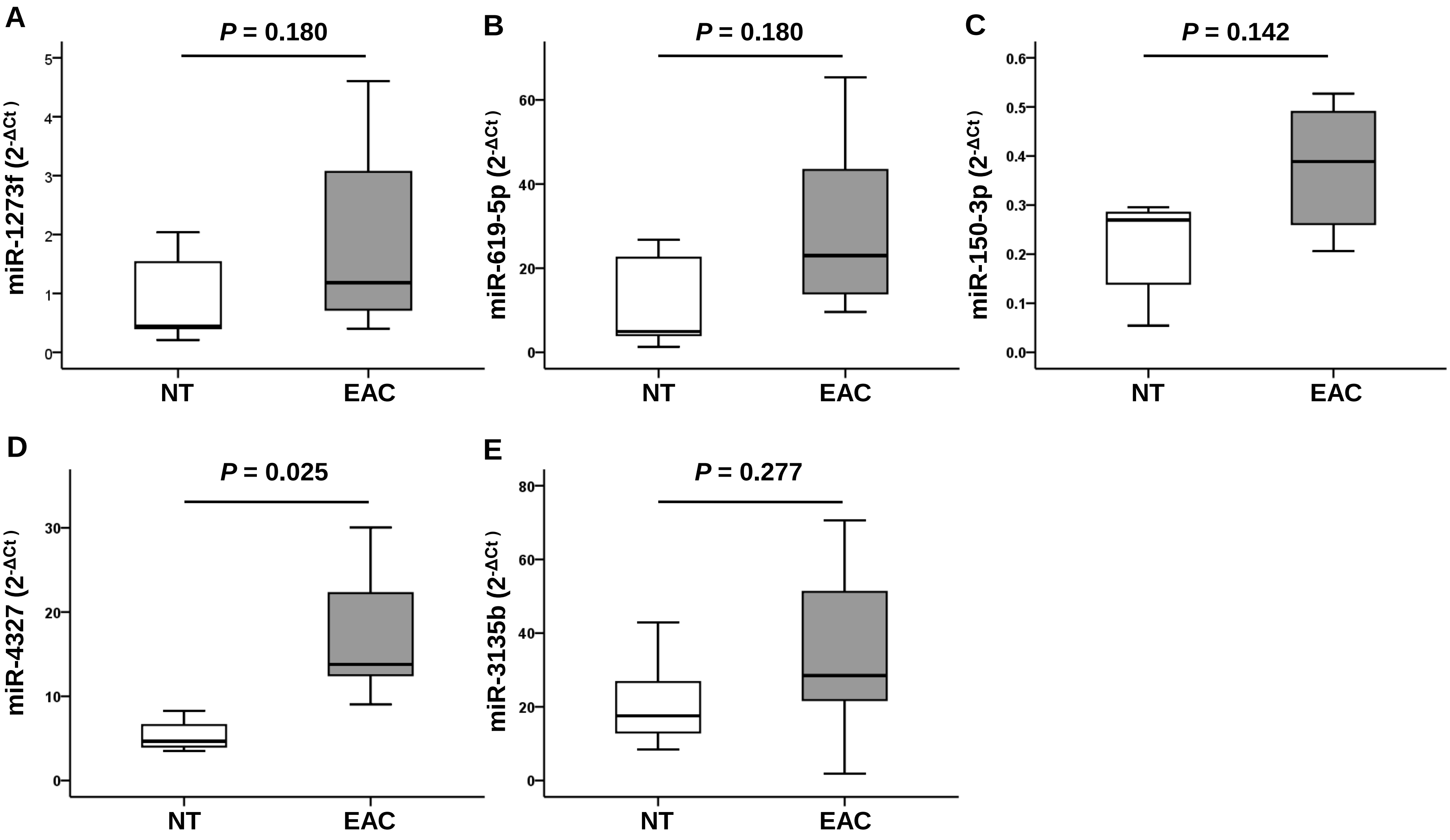

Supplementary Fig. S5. miRNA expression in the esophageal adenocarcinoma and adjacent normal tissues.
A. miR-1273f. B. miR-619-5p. C. miR-150-3p. D. miR-4327. E. miR-3135b.
Data were analyzed using Mann-Whitney U test. (NT, N = 7; EAC, N = 7)
NT, normal tissue; EAC, esophageal adenocarcinoma

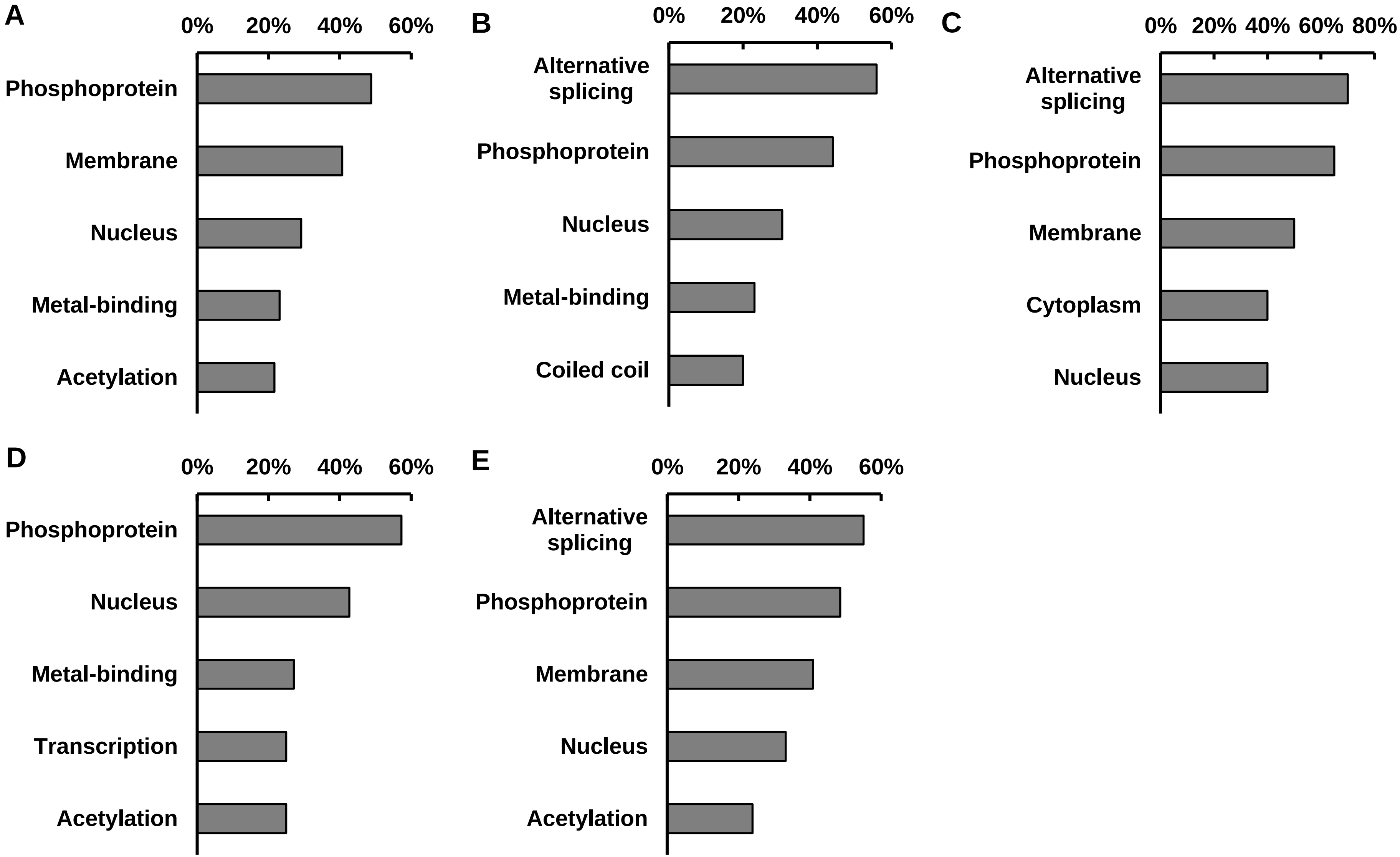

Supplementary Fig. S6. Functional annotation analysis.
A. miR-1273f. B. miR-619-5p. C. miR-150-3p. D. miR-4327. E. miR-3135b.
Functional annotation analysis using the target genes of each miRNA. If there were more than five terms enriched in each category, the top five terms were selected.
